# Incidental branch retinal artery occlusion on optical coherence tomography angiography presenting as segmental optic atrophy in a child: a case report

**DOI:** 10.1186/s12886-017-0653-6

**Published:** 2017-12-19

**Authors:** Ji Hyung Choi, Hee Kyung Yang, Ji Eun Lee

**Affiliations:** 1 Department of Ophthalmology, Maryknoll Medical Center, 121, Junggu-ro, Jung-gu, Busan, 48972 South Korea; 2 Department of Ophthalmology, Seoul National University College of Medicine, Seoul National University Bundang Hospital, #166, Gumiro, Bundang-gu, Seongnam, Gyeonggi-do 463-707 South Korea

**Keywords:** Segmental optic atrophy, Childhood optic atrophy, Branch retinal artery occlusion, Spectral domain optical coherence tomography, Spectral domain optical coherence tomography angiography

## Abstract

**Background:**

Retinal artery occlusion is extremely rare in the pediatric population and most patients have risk factors. We report a case of a healthy child with segmental optic atrophy, complicated by incidental branch retinal artery occlusion (BRAO).

**Case presentation:**

A 10-year-old boy who had a history of his mother’s gestational diabetes presented with an inferonasal visual field defect in the left eye. His best-corrected visual acuities were 20/20 in both eyes (OU). Fundoscopic examination revealed segmental pallor of the left optic disc, thinning of the superotemporal rim, a relative superior entrance of the central retinal artery and superior peripapillary scleral halo. Fluorescein angiography showed patchy filling delays in the corresponding disc area without retinal vascular abnormalities. Spectral domain optical coherence tomography (SD OCT) via automated segmentation analysis demonstrated sectoral absence of the ganglion cell layer and retinal nerve fiber layer with thinning of the inner plexiform layer, inner nuclear layer and outer plexiform layer in the corresponding retina. OCT angiography (OCTA) showed focal attenuation of superficial and intermediate/deep capillary plexuses in the corresponding areas. Systemic evaluation was unremarkable. The patient was diagnosed with segmental optic atrophy caused by incidental BRAO.

**Conclusions:**

Retinal vascular occlusions are rare in childhood, and may present as segmental optic atrophy mimicking congenital anomalies. OCTA allows the detection of previous microvascular abnormalities in the chronic phase. To the best of our knowledge, this is the first report of a child with segmental optic atrophy presumably caused by BRAO, which was documented by SD OCT and OCTA in detail.

## Background

When segmental atrophy of the optic disc is found in children, benign conditions such as isolated superior segmental optic hypoplasia (SSOH) are mostly considered, particularly in healthy children of an insulin dependent diabetic mother [1]. Rarely, branch retinal artery occlusion (BRAO), isolated or secondary to congenital retinal vascular abnormalities can also cause these similar localized optic nerve changes after an acute insult in childhood [2–4]. Of note, the chronic phase of retinal arterial occlusion shows resultant thinning and atrophy of the retinal layers, corresponding to the area of acute lesions [5]. Recent advances in spectral domain optical coherence tomography (SD OCT) and OCT angiography (OCTA) demonstrate the spectrum of capillary ischemia in retinal arterial occlusive diseases presenting variable involvement of the superficial and intermediate/deep capillary plexuses regardless of disease phase [5]. Herein, we present a pediatric case of incidentally found segmental optic atrophy presumably caused by BRAO, which was documented by SD OCT and OCTA.

## Case presentation

**Fig. 1:**
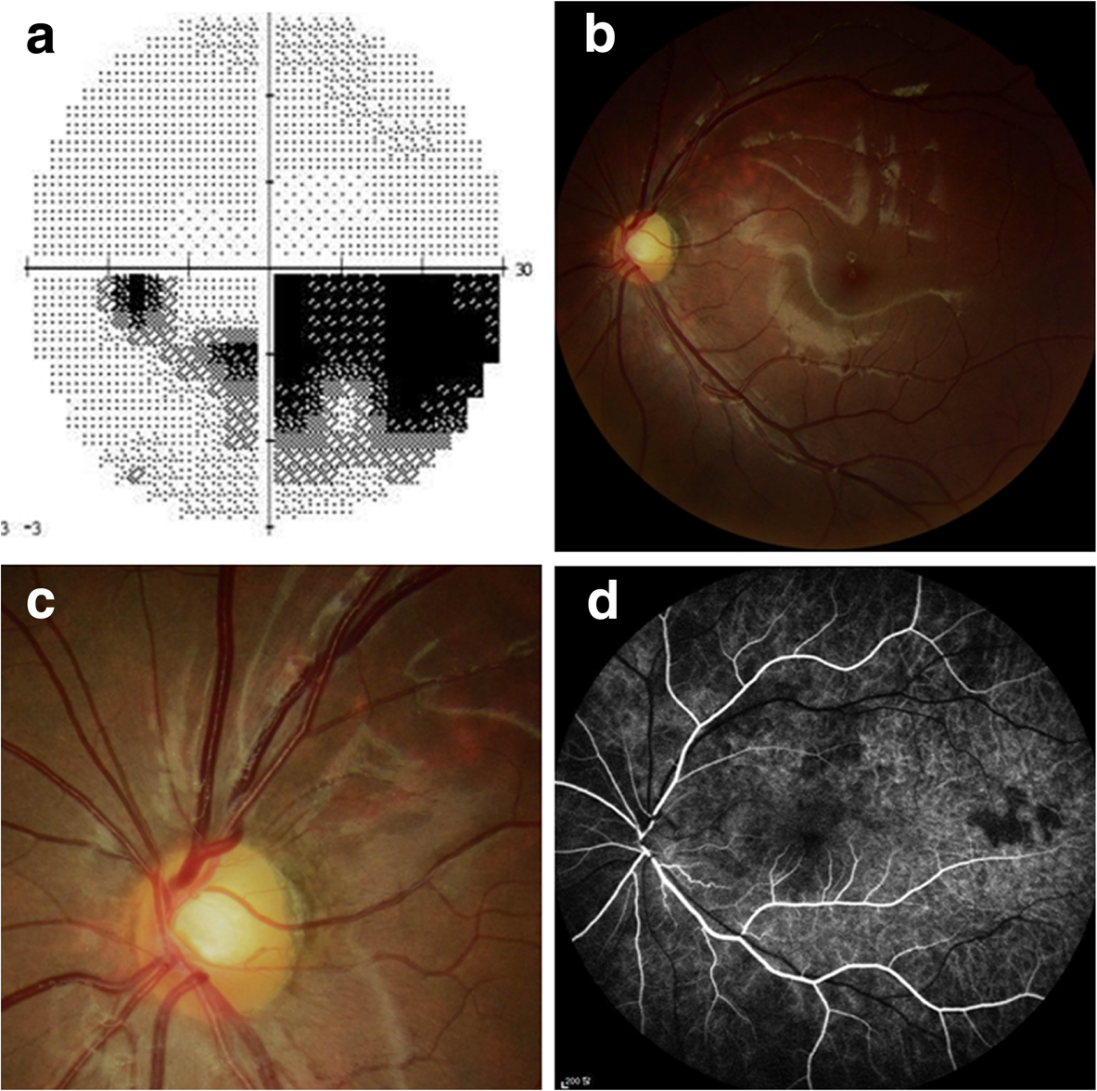
**a** Visual field test shows an inferonasal field defect in the left eye. **b** Fundus photography shows a superotemporal retinal nerve fiber layer defect in the left eye. **c** Optic disc photography shows segmental pallor and rim thinning of the superotemporal quadrant of the optic disc. **d** Fluorescein angiography showed normal retinal vasculatures except microvascular filling delays in the superotemporal disc area

**Fig. 2:**
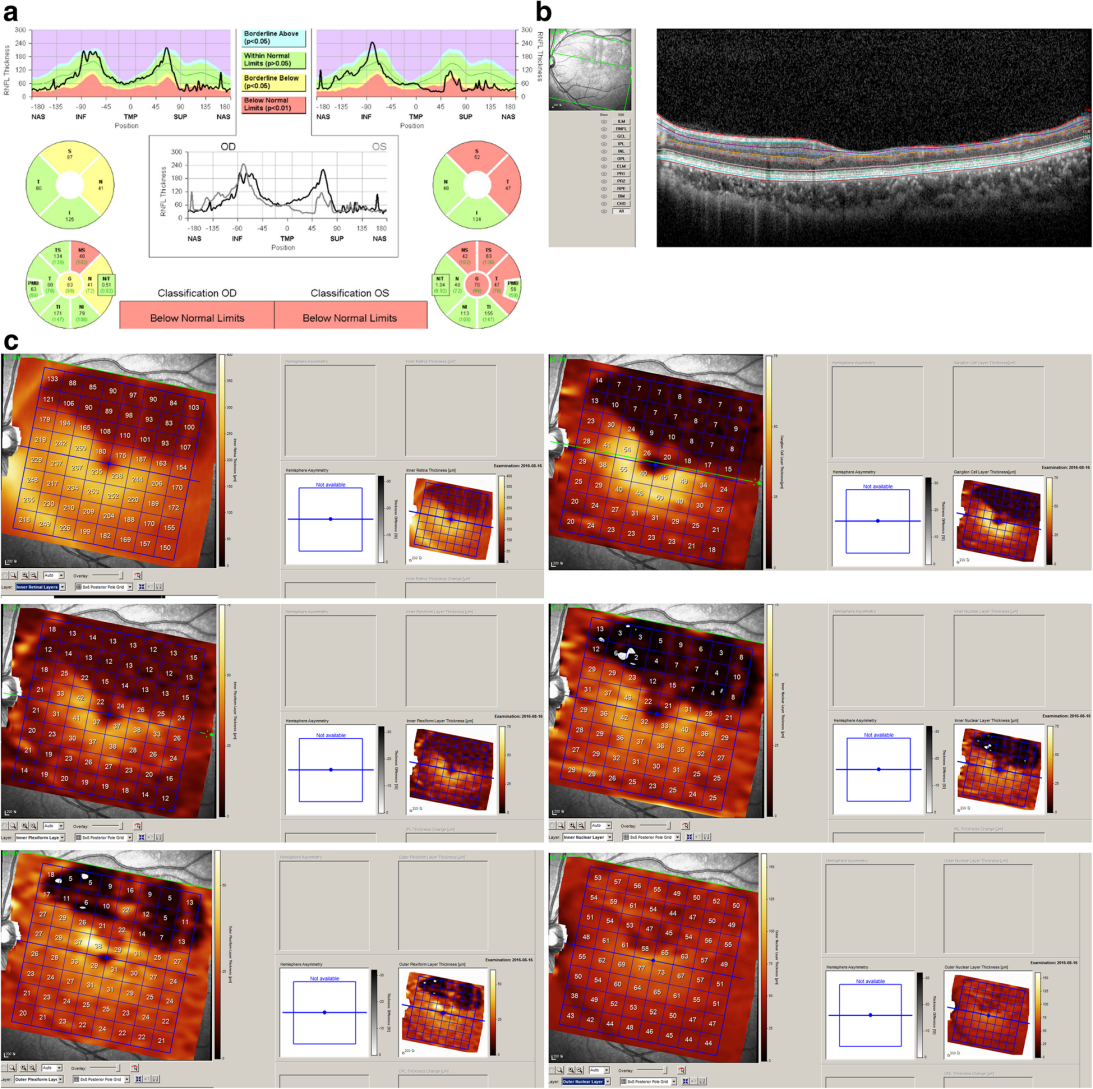
On spectral domain optical coherence tomography (SD OCT), **a** superotemporal peripapillary retinal nerve fiber layer (RNFL) defect was noted with intact papillomacular bundles. **b** The absence of the 3 innermost retinal layers was demonstrated on SD OCT. **c** Automated segmentation analysis showed sectoral absence of the ganglion cell layer (GCL) and RNFL in the corresponding retina as well as marked thinning of the inner plexiform layer (IPL), inner nuclear layer (INL) and outer plexiform layer (OPL)

**Fig. 3:**
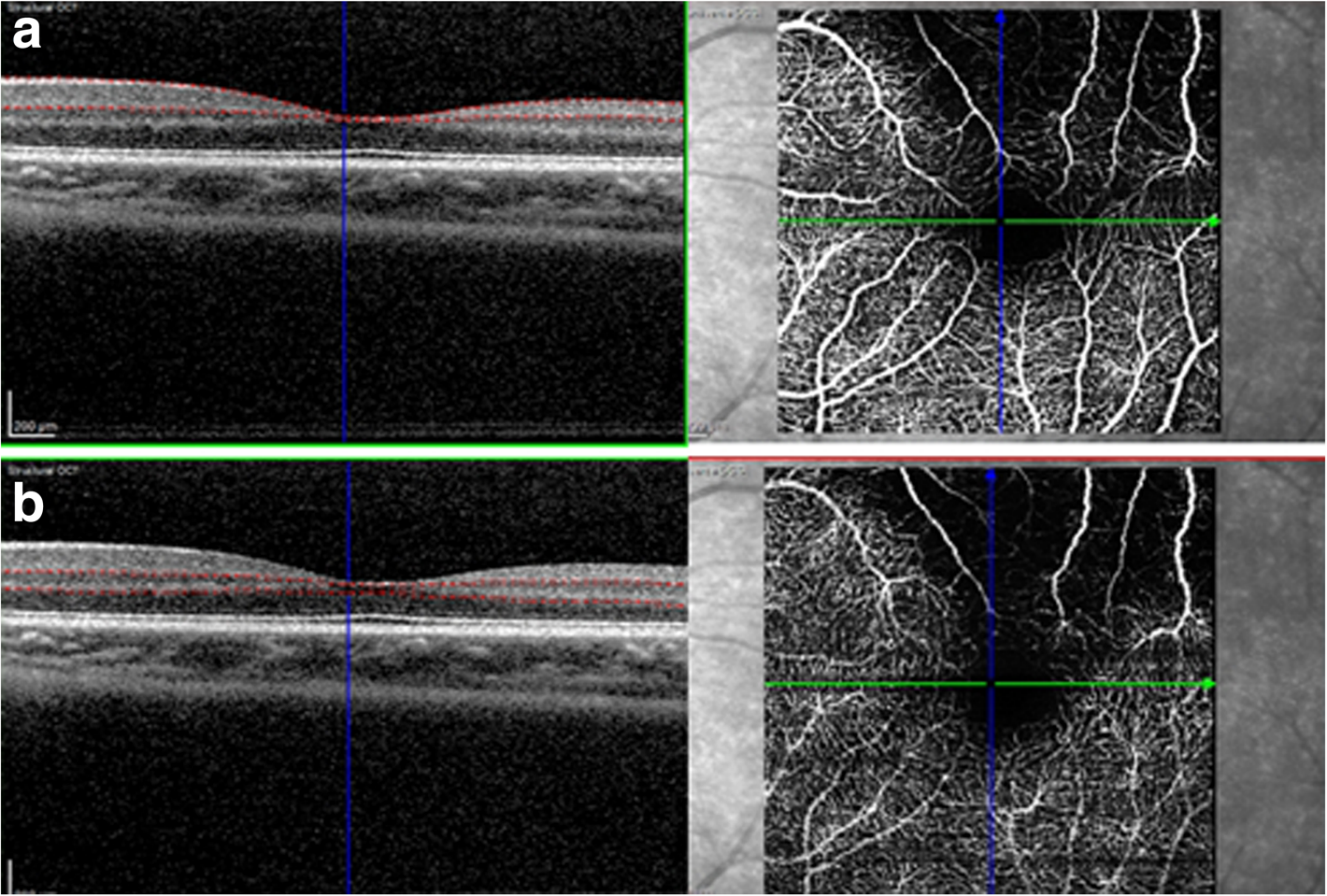
On optical coherence tomography angiography, **a** focal attenuation of superficial and **b** intermediate/deep capillary plexuses were detected in the corresponding areas of retinal thinning

A visually asymptomatic 10-year-old boy presented with an inferonasal visual field defect in the left eye (Fig. 1a). He had no significant medical history other than his mother’s gestational diabetes. His family history was unremarkable for any ocular diseases and his parents showed a normal fundus on examination. His best-corrected visual acuities were 20/20 in both eyes (OU). Pupils were equal, round, and reactive to light without relative afferent pupillary defect. He had normal color vision OU. Detailed fundoscopic examination revealed segmental pallor of the left optic disc, thinning of the superotemporal rim, a relative superior entrance of the central retinal artery and superior peripapillary scleral halo (Fig. 1b and c). Fluorescein angiography showed patchy filling delays in the corresponding disc area but no other retinal vascular abnormality was found (Fig. 1d). SD OCT (Spectralis OCT, Heidelberg Engineering, Heidelberg, Germany) using segmentation analysis showed sectoral absence of the ganglion cell layer (GCL) and retinal nerve fiber layer (RNFL) in the superotemporal quadrant of the retina (Fig. 2a and b). Additionally, marked thinning of the inner plexiform layer (IPL), inner nuclear layer (INL) and outer plexiform layer (OPL) were detected (Fig. 2c). Decreased P100 amplitude in the left eye was observed on visual evoked potential (VEP). OCTA (Spectralis OCT-A, Heidelberg Engineering, Heidelberg, Germany) revealed attenuated superficial (Fig. 3a) and intermediate/deep capillary plexuses (Fig. 3b). The main branches in corresponding well-demarcated lesions were preserved. Consequently, his segmental optic disc pallor and inner retinal hypoplasia were presumed to be caused by an incidental BRAO. To evaluate risk factors for retinal arterial occlusion, laboratory investigations were performed including complete blood count with erythrocyte sedimentation rate, serum lipids, metabolic panel, liver and renal function tests, which were all normal. A thrombophilia screening revealed that homocysteine, protein C, protein S, factor V Leiden assay, antithrombin III, prothrombin time, fibrinogen, lipoprotein A and complement levels were normal. Rheumatoid factor, antinuclear antibodies, lupus anticoagulant, antineutrophil cytoplasmic antibodies, antiphospholipid antibodies, and anticardiolipin antibodies were absent with no evidence of vasculitis. Cardiovascular work-up including echocardiography showed no abnormalities and systolic/diastolic blood pressure were within normal limits.

## Discussion and conclusions

Optic nerve atrophy is the secondary pathogenic endpoint of numerous diseases that cause intrinsic or extrinsic insult to the visual pathway. Although, partial optic atrophy with homonymous visual field defect is characteristic for cortical visual impairment, various congenital lesions involving the retina, optic nerve, chiasm, optic tract, or retrogeniculate pathways as well as acquired conditions such as periventricular leukomalacia also show segmental optic nerve changes [6, 7]. However, our patient had none of these lesions or history of preterm delivery. In this patient, the clinical presentations including good visual acuity, inferior visual field defect pattern, relative superior entry of the central retinal artery, superior RNFL defect, superior disc pallor and a history of maternal diabetes are common features of SSOH. However, to the best of our knowledge, there has been no description about this type of variant involving the inner retinal layers beyond GCL and RNFL in the literature, on the contrary to the typical OCT findings of SSOH featuring thinning of the superior segmental peripapillary RNFL [8]. This newly observed case resembles previously reported retinal changes associated with optic nerve hypoplasia [9]. The sectoral defect pattern in this case was also similar to the report of an optic nerve head pit and focal inner retinal hypoplasia [10]. OCTA images were analogous to the chronic phase of BRAO showing marked decrease of capillary densities with retinal thinning and atrophy [11]. Thus, this case strongly suggests secondary optic atrophy related to BRAO rather than a primary congenital anomaly. Fluorescein angiography may not be helpful in such cases of subacute or chronic phase BRAO, as the blood flow in the retinal arteries are typically reconstituted in a week with resolution of inner retinal edema in 4–6 weeks [12]. However, the inner retinal damage is permanent, leading to atrophy, attenuated retinal microvasculature and optic disc pallor [12]. During the chronic stage of RAO, inner retinal thinning or atrophy due to ischemia may be pathognomonic [12].

Retinal artery occlusion is an extremely rare condition in the pediatric population and most patients have some detectable risk factors [13]. Surprisingly, our patient was hematologically normal, free of cardiac diseases or migraine histories and showed no vascular abnormality such as retinal macrovessels [2] or prepapillary vascular loops [3]. To date, 2 reports exist of idiopathic BRAO in children [14, 15]. One case had a persistent visual field defect, as found in our patient [14]. Although retinal artery occlusion is rare in children, the recognition of segmental optic disc abnormality coexisting with corresponding retinal thinning and attenuation of microvasculature should prompt questioning for this condition and the need for proper investigative studies. This case highlights that pediatric BRAO can clinically mimic congenital abnormalities such as segmental optic nerve hypoplasia or atrophy, especially in the chronic phase of the disease. The ability of OCTA to visualize fine microvascular abnormalities at the superficial and deep levels allows the detection of these masquerading conditions that may arise as a consequence of previous retinal artery occlusions.

## Abbreviations

BRAO: Branch retinal artery occlusion
GCL: Ganglion cell layer
INL: Inner nuclear layer
IPL: Inner plexiform layer
OCTA: Optical coherence tomography angiography
OPL: Outer plexiform layer
OU: Both eyes
RNFL: Retinal nerve fiber layer
SD OCT: Spectral domain optical coherence tomography
SSOH: Superior segmental optic hypoplasia
VEP: Visual evoked potential
